# Surface bioactivation through the nanostructured layer on titanium modified by facile HPT treatment

**DOI:** 10.1038/s41598-017-04395-0

**Published:** 2017-06-23

**Authors:** Zhijun Guo, Nan Jiang, Chen Chen, Songsong Zhu, Li Zhang, Yubao Li

**Affiliations:** 1 The Research Center for Nano Biomaterials, Analytical & Testing Center, Sichuan University, Chengdu, 610064 China; 2 State Key Laboratory of Oral Diseases, National Clinical Research Center for Oral Disease, West China Hospital of Stomatology, Sichuan University, Chengdu, Sichuan 610064 China

## Abstract

Facile fabrication of nanostructured surface is of great importance for the use of titanium (Ti) implants in biomedical field. In this study, a low-cost and easy-to-operate method called HPT (hydrothermal & pressure) here has been developed and used to fabricate the expected nanostructured surface on Ti substrates. The effects of experimental parameters on the morphology of Ti surface were investigated and characterized. The results indicated that by altering the hydrothermal pressure, NaOH concentration and treating time, surface nanostructure like nanopetals or nanoflakes could be formed on the surface of Ti substrates. The orthogonal experiments were conducted to demonstrate the optimized operation conditions. A formation mechanism of the nanostructured titanate layer was proposed, revealing that the nanostructured layer could be formed via a special upward and downward co-growth manner. *In vitro* cell culture showed that the HPT treated Ti substrates, especially the T-10 sample, could greatly enhance the cell-material interactions, i.e. the cell proliferation and differentiation, focal protein adhesion, and osteogenic factor expression. The HPT method paves a new way to modify the surface of Ti implants with better bioactivity and promising prospect for future biomedical applications.

## Introduction

Titanium (Ti) and its alloys have been the most commonly employed implant materials for dental and orthopaedic applications owing to their excellent mechanical property, corrosion resistance in body fluid, and good biocompatibility with human tissues^1–5^. Ti is a relatively light metal that possesses good ductility, high tensile strength and an elastic modulus matching to human cortical bone. However, as a bioinert material, titanium implants without surface modification generally lack osseointegration with the surrounding bone tissue, leading to a short lifespan or even implantation failure^6^. So, suitable surface treatment for Ti implants is essential to enhance their integration with bone tissue. It has been reported that the formation of a porous structure on Ti substrate was proved to be very effective in providing nucleation sites for Ca-P precipitation, and in integration with surrounding bone tissue^2^. In recent years, a nanostructured surface on Ti substrate is also expected to further improve its bioavailability and bioreactivity^7, 8^, and to enhance the biological response of protein attachment, cell adhesion and growth, and bonding with bone tissue. But it is still a challenge that how to fabricate a nanostructured porous surface on titanium using an easy and cost-effective method^9, 10^. Therefore, we still need to find a promising route to achieve a nanostructured porous surface and demonstrate its effectiveness in endowing high bioactivity.

In literatures, various methods have been used to prepare the expected surfaces on Ti and its alloys, such as chemical treatment^11^, sol-gel method^12^, chemical vapor deposition^13^, anodization^14^, hydrothermal (HT) method^15^ and electro-deposition^16^, plasma or microarc oxidation^17–19^. Among them, chemical treatment in alkali solutions is a strong contender to produce a nanoscaled network structure on the surface of Ti and its alloys. Wu *et al*.^20^. prepared a nanostructured titanate layer on Ti substrate in 10 M NaOH solution at 60 °C for 240 h and found that the layer displayed super-hydrophilic ability, favoring the rapid deposition of hydroxyapatite (HA) and accelerating the cell’s attachment and proliferation. The hydrothermal (HT) method is another approach to fabricate nanostructured titanate surface by soaking Ti or Ti alloy in alkali solution, which showed high crystallinity due to the dissolution- recrystallization process^21^. By treating Ti substrate in a concentrated NaOH solution at 110 °C for 90 h, Yu *et al*.^22^. Obtained polycrystalline titania nanotubes with an outer diameter of 9−12 nm and a length of 100−600 nm. However, both the chemical treatment and the HT method require a long reaction time (about 3 days) to achieve the nanostructured titanate layer on Ti substrates^23^, that might be high-cost, time consuming and difficult to operate.

For HT method, it has confirmed that the use of a higher pressure could often generate unexpected effect in preparation of new compounds with higher growth rate due to its higher energy than other usual methods^24, 25^. And an increased reaction temperature could also result in the formation of multi-layer nanostructure instead of nanotubes^26^. Therefore, in this study, we designed a rapid fabricating method to achieve a nanostructured porous titanate layer on the surface of Ti substrate by selecting relatively high reaction pressure and temperature in HT treatment. A pressurized reactor with 147 KPa (about 0.15 MPa) pressure and 120 °C reaction temperature was used to treat Ti substrates in NaOH solution (we named the high pressure and temperature hydrothermal method as HPT in this paper). The relationship and function between NaOH concentration and Ti surface morphology were investigated in detail to find the appropriate concentration of NaOH solution in treating Ti substrates. Some Ti samples with special or different surface structure were chosen to evaluate their influence on behaviors of BMSCs *in vitro*.

## Materials and Methods

### Surface modification of Ti substrates

Commercially pure Ti substrates (Ti > 99.5%, Baoji Titanium industry Co. Ltd., China) were cut into rectangular plates of 10 × 10 × 1 mm^3^. Firstly, these plates were chemically washed with a mixture solution of HF: HNO_3_: H_2_O_2_ at a volume ratio of 1:3:5 for 5 min to remove the naturally formed oxide layer on the Ti surface. Then, they were ultrasonicly washed with acetone, 2-propanol, alcohol and deionized water for 30 min separately and dried in a vacuum freeze drier. Afterwards, the cleaned Ti plates were immersed into 80 ml NaOH aqueous solutions with the concentration of 0.5 M, 1 M, 3 M, 5 M, 7.5 M, 10 M 12.5 M, 15 M and 18 M respectively, and put in a 200 mL Teflon-lined autoclave for hydrothermal treatment at 0.15 MPa and 120 °C for 10, 30, 60, 120, 240 and 480 min. Finally, these treated Ti plates were gently washed with deionized water thoroughly and dried in air at room temperature for 24 h. The obtained samples were stored for further use. The untreated pure Ti sample, and 3M-Ti, 10M-Ti, 12.5M-Ti samples treated for 240 min, were designated as PT, T-3, T-10 and T-12.5, respectively.

### Characterization

The Ti plates treated at different conditions were observed by scanning electron microscopy (SEM, JSM-6510LV, JEOL). Their microscopic morphologies were also observed by atomic force microscope (AFM, MFP-3D Asylum Research, Santa Barbara, CA). The crystallographic structures of the samples were detected with X-ray diffraction (XRD, RINT-2000, Rigaku, Japan). The surface elements were analyzed by X-ray photoelectron spectroscope (XPS, VG Scientific, ESCALAB 250, Sussex, UK) performed with Al Kα (hν = 1486.6 eV) as the X-ray source. The wettability and surface free energy of the samples were analyzed using contact angle goniometer (TL101, Biolin Scientific AB). The concentration of Ti released into alkaline solution was measured using an inductive coupled plasma atomic emission spectrometer (ICP-AES, IRIS Adv). The morphology and composition of Ca-P precipitates deposited on nanostructured titanate layer were analyzed by SEM and its coupled energy dispersive spectrometer (EDS).

### *In vitro* cell behaviour

#### Cell isolation and culture

Bone marrow stromal cells (BMSCs) were isolated from the femurs of 2-week-old male Sprague-Dawley rats (Animal Research Center, Sichuan University, China). All animal care and experiments were guided in line with the standards of the Animal Research Committee of the West China School of Stomatology, Sichuan University, and conducted in accordance with international guidance on animal welfare. BMSCs were cultured in alpha-modified Eagle’s medium (α-MEM, Gibco, Gaithersburg, USA), which was supplemented with 10% fetal bovine serum (FBS, Gibco, Gaithersburg, USA), 50 mg/L ascorbic acid (Sigma-Aldrich, St. Louis, USA), 10 mM Na-β-glycerophosphate (Sigma-Aldrich, St. Louis, USA), 10^−8^ M dexamethasone (Sigma-Aldrich, St. Louis, USA), and 1% penicillin-streptomycin antibiotic antimycotic solution (Invitrogen, Carlsbad, USA). The cells were incubated in a humidified atmosphere of 95% air and 5% CO_2_ at 37 °C.

#### Cell-material interaction

PT, T-3, T-10 and T-12.5 were sterilized and placed in the wells of 24-well culture plate, respectively. The cells at passage 3 were seeded onto these Ti samples with a density of 5 × 10^4^ cells/ml, and after 2 h of culture, the Ti samples with attached cells were fixed with 2.5% glutaraldehyd and dehydrated in a graded series of ethanol (20%, 40%, 60%, 75%, 90%, 100%). The Ti samples were dried by a EMS 850 critical point dryer (Electron Microscopy Science Co., Hillsboro, USA), and sputter-coated with a palladium layer using a JFC-1600 ion sputtering apparatus (Electronics Co., Ltd, Saitama, Japan) for SEM examination. Cell proliferation on PT, T-3, T-10 and T-12.5 samples was determined using a 3(4,5-dimethylthiazol-2-yl)−2,5-diphenyl tetrazolium bromide assay (MTT, M-2128, Sigma, St. Louis, MO, USA) after culturing for 1, 3, 5, 7, and 9 days. Meanwhile, the cells seeded on all Ti samples after incubation for 7, 10, and 14 days were fixed by 4% paraformaldehyde and stained with Alkaline phosphates (ALP) kit (Beyotime, Shanghai, China) and the ALP activity was obtained through measurement of optical density using the spectrophotometer at 405 nm.

#### Focal adhesion staining

After cell seeding on PT, T-3, T-10 and T-12.5 samples for 24 h, the BMSCs were stained to show their F-actin filaments and vinculin, using the Actin Cytoskeleton and Focal Adhesion Staining Kit (FAK100) (Millipore) according to the manufacturer’s instructions. The cells were also stained with Alexa Fluor 647-conjugated phalloidin and FITC-conjugated anti-vinculin antibodies to detect cytoskeletal and focal adhesion proteins, respectively. The stained cells were observed under a confocal microscope (LSM700, Carl Zeiss, Oberkochen, Germany).

#### Quantitative real-time polymerase chain reaction

Quantitative real-time polymerase chain reaction analysis (qRT-PCR) was performed to examine the expression of various osteogenic factors, including runt-related transcription factor 2 (Runx2), osteocalcin (OCN), osteopontin (OPN), and collagen type I (COL-1) (shown in Table 1). After incubation with the disk-shaped PT, T-3, T-10 and T-12.5 samples for 3, 7, and 14 days, the cells were detached by 0.25% trypsin-1 mM EDTA (Gibco BRL, Gaithersburg, MD, USA). Total RNA was extracted using TRIzol reagent (Invitrogen, Carlsbad, CA, USA) and reversely transcribed to cDNA employing a first-strand cDNA synthesis kit (Takara, Shiga, Japan). The mRNA expression of the above-mentioned osteogenic factors was determined using a StepOnePlus Real-Time PCR System (Applied Biosystems, Foster City, CA, USA). The GAPDH was used as the internal RNA control, and the relative expression levels of the osteogenic factors were obtained according to the 2^−ΔΔCt^ method.

**Table 1:** Real-time polymerase chain reaction primers used in this study.

Target gene	primer
COL-1	
rCOL-1F	GCTGGCAAGAATGGCGAC
rCOL-1R	AAGCCACGATGACCCTTTATG
OCN	
rOCNF	GGAGGGCAGTAAGGTGGTGA
rOCNR	ACGGTGGTGCCATAGATGC
OPN	
rOPNF	AACAGTATCCCGATGCCACA
rOPNR	TGGCTGGTCTTCCCGTTG
RUNX-2	
rRUNX-2F	CAGGCGTATTTCAGATGATGACA
rRUNX-2R	TAAGTGAAGGTGGCTGGATAGTG
GAPDH	
rat actin f	CCCATCTATGAGGGTTACGC
rat actin r	TTTAATGTCACGCACGATTTC

#### Data anaysis

The data were treated with a SPSS 22.0 statistical package (SPSS 22.0, USA) and analyzed by ANOVA software for multiple comparisons. The difference was considered statistically significant for p < 0.05.

## Results and Discussion

### Surface morphology versus hydrothermal conditions

**Figure 1:**
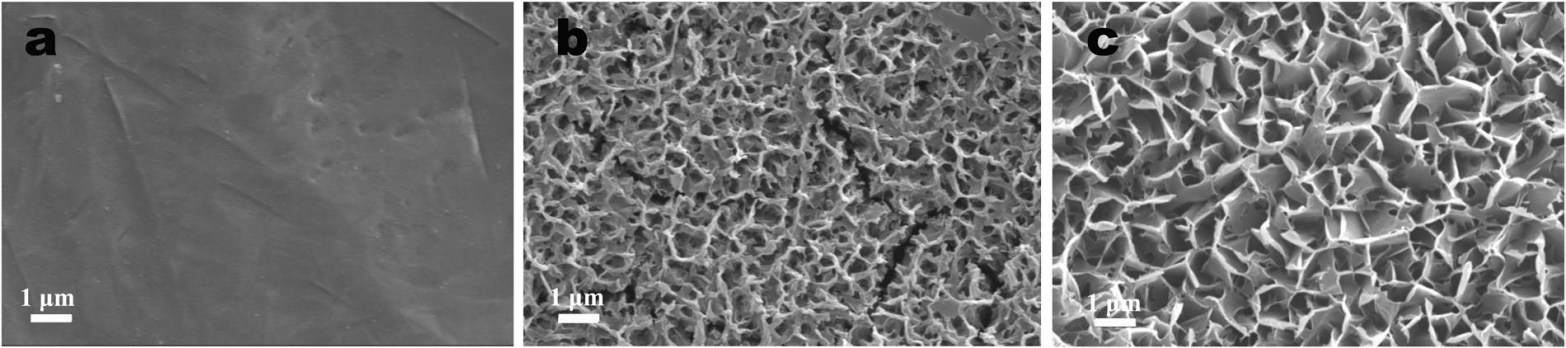
SEM images of surface morphologies for Ti plates: (**a**) pure Ti surface, (**b**) treated at 100 °C for 4 h in 10 M NaOH solution under atmospheric pressure and (**c**) treated at 120 °C for 240 mins in 10 M NaOH solution under 0.15 MPa.

It can be seen that the untreated Ti plate shows a smooth surface relatively (Fig. 1a). After HPT treatment in 10 M NaOH solution for 240 mins under different temperature and pressure, the Ti plate treated at 100 °C and atmospheric pressure presents a nanoscale network on the surface with a pore size of 300–500 nm (Fig. 1b), while the Ti plate treated at 120 °C and 0.15 MPa displays a uniform 3D flake-like nanostructure (Fig. 1c) with the pore size ranging from 600 to 900 nm. For a single flake in Fig. 1c, the flake width is about 400–800 nm and the flake thickness is less than 50 nm. It indicates that the hydrothermal temperature and pressure have obvious effect on the surface morphology or surface nanostructure of the Ti plates; higher temperature and pressure could provide an additional driving force for the nucleation and growth of surface nanostructured layer in alkali solution and make the flakes grow faster.

**Figure 2:**
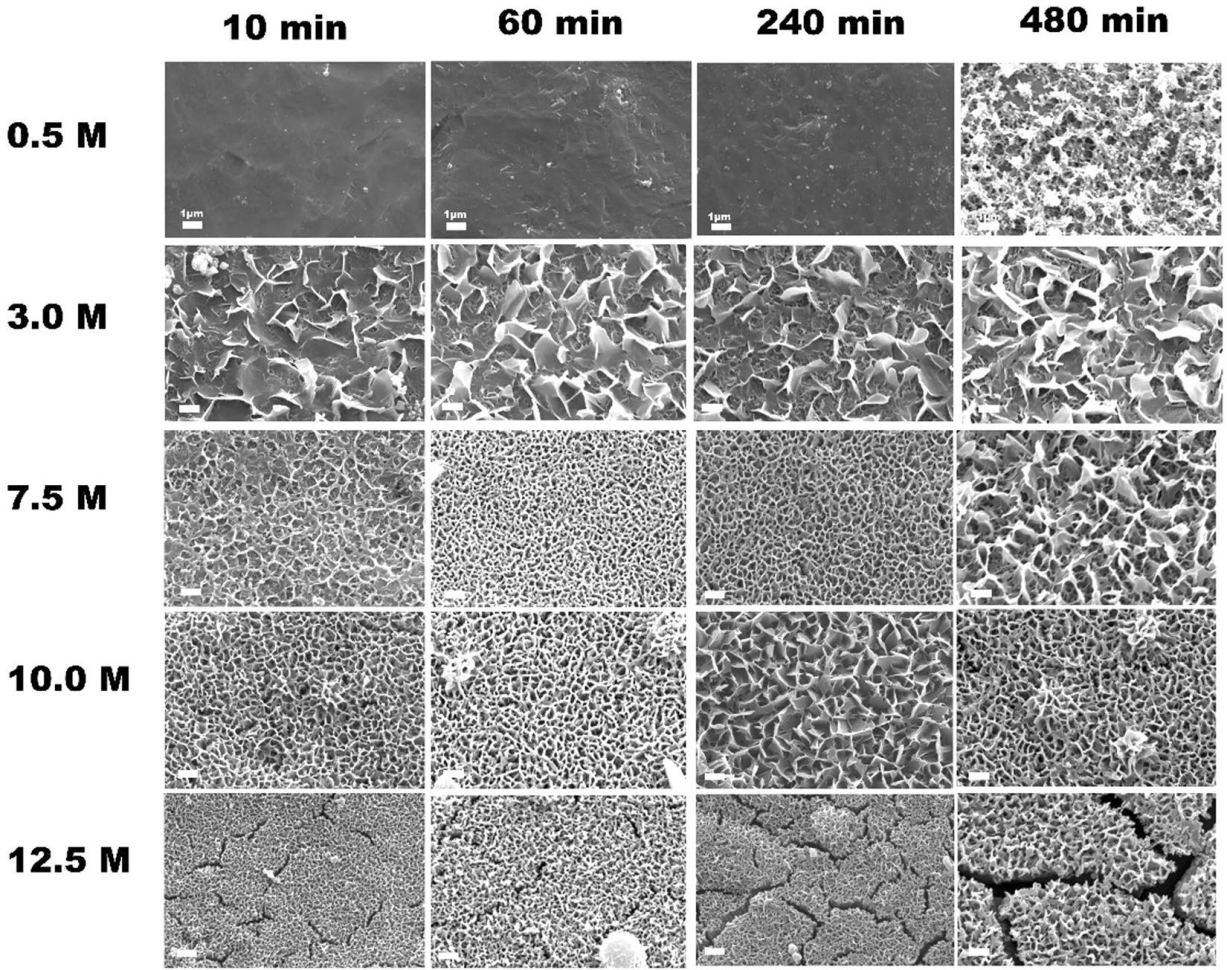
SEM images of Ti surfaces treated by HPT method at 120 °C for 10, 60 and 240 min in NaOH solution of different concentrations. Bar = 1 µm.

Figure 2 shows the influence of NaOH solution concentration and treating time on the morphologies of Ti samples. The surface of Ti plate treated in 0.5 M NaOH solution has almost no apparent change, and still keeps relatively smooth until 240 min When treated in 3 M NaOH solution, a petal-like structure, but not a uniform interconnected porous structure, has formed on the Ti surface at all time points. The width of these petals is from 800 nm to 1.3 µm and the thickness is less than 50 nm. When treated in 7.5 M NaOH solution, nanoscale network structure can be obtained on the surface of Ti plate, the pore size is from 100 nm and 400 nm after treating for 240 min. By further increase to 10 M of the NaOH concentration, the Ti surface changes from nanoscale network to flake-like morphology with larger pore size and an interconnected porous structure. After treating in 12.5 M NaOH solution for 10, 60 and 240 min, a surface layer with cracks and smaller pore network can be observed on Ti plate. When the NaOH concentration is more than 15 M, there are no special structure features present, and just a little round granules are dotted on the Ti surface (see Fig. S1 in Supporting Information). These results demonstrate that the NaOH solution concentration and the treating time significantly affect the surface morphology of the Ti plates. A preferred NaOH concentration and treating time can be determined as 7.5–10.0 M and 240 min (4 h) for the HPT method to fabricate flake-like nanostructured porous surface on Ti substrate. Four samples with different surface morphologies, i.e. untreated PT, and treated T-3, T-10, T-12.5 for 240 min, are chosen to evaluate their *in vitro* cell-material interaction.

**Figure 3:**
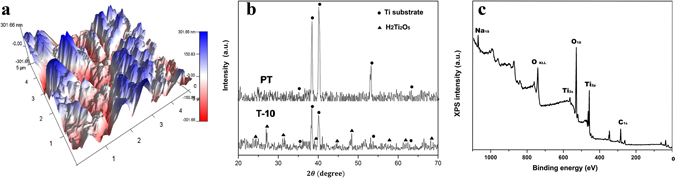
Surface roughness and composition of T-10 sample treated at 120 °C in 10 M NaOH solution for 4 h: (**a**) AFM micrograph, (**b**) X-ray diffraction patterns and (**c**) wide-range survey scan of XPS spectrum.

Figure 3a shows that the treated T-10 sample has a surface roughness of about 112.7 nm, which is much higher than that of untreated PT sample (about 20.7 nm). Compared with the surface free energy (SFE, 39.03 mJ/m^2^) of PT sample (Table 2), the treated T-10 sample has an increased SFE value of about 70.46 mJ/m^2^. The water contact angle is about 62.7° on the surface of untreated Ti plate, whereas it decreases significantly to about 17.9° for T-10 sample. The results indicate that the flake-like nanostructured layer can markedly improve the surface roughness and hydrophilicity of Ti substrate. It has been reported that the microtopography, surface roughness and wettability of Ti substrate have great impacts on the attachments of proteins or biomacromolecules, which in turn will determine the rate of cell proliferation and influence the osteointegration of Ti implants^27, 28^. Zhang *et al*. also reported that both the roughness and the hydrophilicity of Ti surface could promote the proliferation of osteoblast-like cells^29^. In addition, the flake-like nanostructured surface of T-10 sample has proved to be very effective in enhancement of the surface bioactivity of Ti substrate by providing more nucleation sites and promoting the Ca-P precipitation (see Figs S2, S3 in Supporting Information).

**Table 2:** Contact angle (*θ*) and surface free energy (SFE) of untreated PT sample and treated T-10 sample.

sample	*θ* (water) (deg)	*θ* (diiodomethane) (deg)	SFE (mJ/m^2^)
PT	62.71 ± 2.87	41.96 ± 0.68	39.03
T-10	17.89 ± 2.18	23.72 ± 3.31	70.46

The XRD pattern of PT sample in Fig. 3b shows the sharp characteristic peaks of untreated Ti substrate, while the treated T-10 sample only presents poorly crystallized diffraction peaks. However, some characteristic peaks belonging to H_2_Ti_2_O_5_•H_2_O can be found in T-10 sample, when comparing with XRD database (JCPDS No. 47–124) and those reported in previous research^20^. Although the peaks assigned to Na_2_Ti_2_O_5_•H_2_O are difficult to distinguish because of possible overlapping with H_2_Ti_2_O_5_•H_2_O or too weak to be clearly defined, but Na_2_Ti_2_O_5_•H_2_O is regarded to be definitely existed. Kim *et al*. reported that the treated porous layer on Ti substrate obtained in NaOH solution certainly contained alkali titanates, and should mainly consist of an amorphous Na_2_Ti_2_O_5_•H_2_O^30^. It has been found that the crystallized water molecules could broaden the diffraction peaks and lower their intensities^30, 31^. The broadening of these peaks can also originate from a change in the average crystallite size, the introduction of lattice defects and/or an increase in the internal strain. Therefore, the increase in surface hydrophilicity of the treated T-10 substrate should be related to the chemical structure of sodium titanate (Na_2_Ti_2_O_5_) which could easily absorb water in the environment.

As shown in the XPS spectrum (Fig. 3c, Fig. S4), the major elements on the treated T-10 surface are Ti, O and Na, and the C_1s_ peak at 284.6 eV is often used as a reference binding energy for calibration. Based on this, we deduced that the flake-like nanostructured layer should be mainly composed of Na_2_Ti_2_O_5_•H_2_O, and the ion exchange between Na^+^ and H^+^ in water might result in the existence of H_2_Ti_2_O_5_•H_2_O. It is still debated in literatures for the final products of Ti substrates after hydrothermal treatment in NaOH solution^32–34^. Several possible compositions have been proposed, including TiO_2_, Na_2_Ti_9_O_19_, Na_2_Ti_6_O_13_, Na_2_Ti_2_O_4_(OH)_2_, Na_2_Ti_3_O_7_•nH_2_O, H_2_TiO_3_O_7_, H_2_Ti_2_O_5_, H_x_Ti_2−x/4_□_x/4_O_4_(□ = vacancy), etc refs 32, 34–40. Bavykin *et al*. reported that Ti substrate treated in alkali solution was a complex process involving the formation of several intermediate structures participating in sequential chemical reaction^25^. Riss *et al*. revealed that Na_2_Ti_3_O_7_ nanowires could turn into H_2_Ti_3_O_7_ nanotubes by washing with diluted HCl^41^. The Na_2_Ti_3_O_7_-like nanocrystals were considered to be unstable and just existed as an intermediate phase. Hence, we conclude that the nanostructured layer on the Ti surface is composed of both H_2_Ti_2_O_5_•H_2_O and Na_2_Ti_2_O_5_•H_2_O.

### Formation mechanism of nanostructured titanate layer

**Figure 4:**
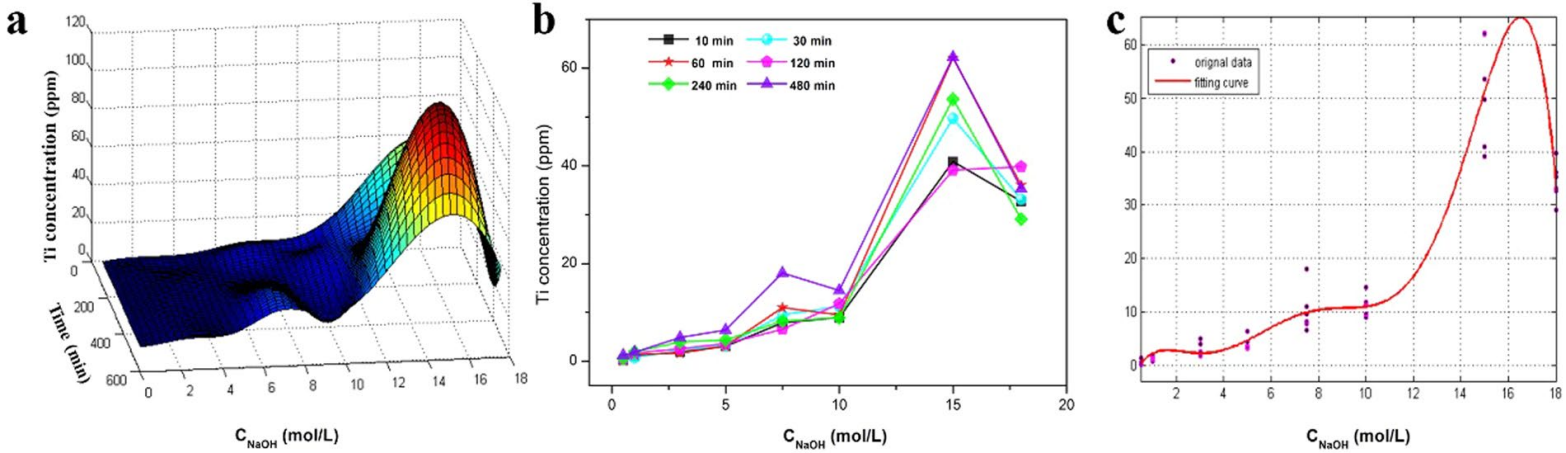
The 3D graph of Ti concentration as a function of NaOH concentration and treating time (**a**), and the functional relationship of Ti concentration vs. NaOH concentration (**b**), (**c**) fitting curve of Ti concentration vs. NaOH concentration.

As observed in Fig. 2, various surface morphologies could be formed on the surface of Ti substrates after HPT treatment in different NaOH solutions for different time. By investigating the Ti concentration in solution, we may indirectly determine the effects of NaOH concentration and treating time on the surface of Ti substrate. An orthogonal experiment was conducted with a NaOH concentration ranging from 0.5 M to 18 M for a period of 10 min to 480 min. It can be seen from the 3D graph in Fig. 4a that the Ti concentration in solution generally rises with the increase of NaOH concentration for all treating time point (Fig. 4b). There are two obvious Ti concentration peaks during the change of NaOH concentration from 0.5 M to 18 M, the small peak covers a NaOH range of 5–10 M and the large peak covers the range of 10–18 M. The small peak range corresponds very well to the preferred NaOH concentration (7.5–10.0 M) determined by SEM observation in Fig. 2, during which the treating time seems the second predominant factor. This may suggest that a NaOH concentration lower than 5 M (i.e. only small amount of Ti leaves the surface of Ti plate during HPT treatment) or more than 10 M (i.e. large amount of Ti leaves the surface of Ti plate) is not beneficial to the obtaining of the desired surface nanostructured layer. In other words, the formation of surface titanate layer is strongly related to both the NaOH concentration and the Ti exchange between the surface and solution (dissolution and deposition) during HPT treatment. Bavykin *et al*. also suggest the importance of Ti concentration in alkaline solution in the surface modification of titanium substrate^42^, which is in coincidence with the result obtained in this study. To establish a relationship between the dissolved Ti concentration (C_Ti_) in solution and the observed morphology of Ti surface (i.e. corresponding to the NaOH concentration, C_NaOH_), we used polynomial function to fit the data (Fig. 4c) and obtained an approximate equation as the following:

1 $${C}_{Ti}=\sum _{i=0}^{6}{a}_{i}{C}_{NaOH}^{i}$$

Where *a*_*i*_ is a constant with 95% confident bounds, in which a_0_ = −4.336, a_1_ = 12.07, a_2_ = −7.322, a_3_ = 1.946, a_4_ = −0.2396, a_5_ = 0.0136, a_6_ = −2.87 e^−^4. According to the equation and the observed morphologies in Fig. 2, we can conclude that nanopetals formation on Ti surface corresponds to a dissolved Ti concentration of 3~5 μg∙mL^−1^ in NaOH solution, while the flake-like nanostructured layer relates to the dissolved Ti concentration range of 5~15 μg∙mL^−1^.

**Figure 5:**
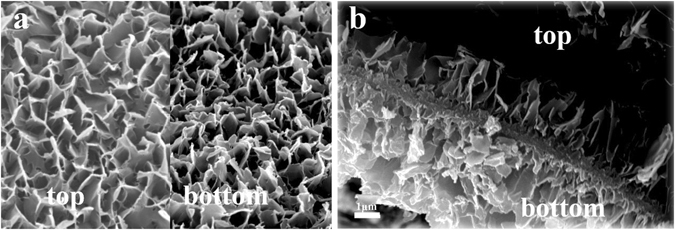
SEM images of flake-like nanostructured layer (**a**) top and bottom view, (**c**) cross section.

**Figure 6:**
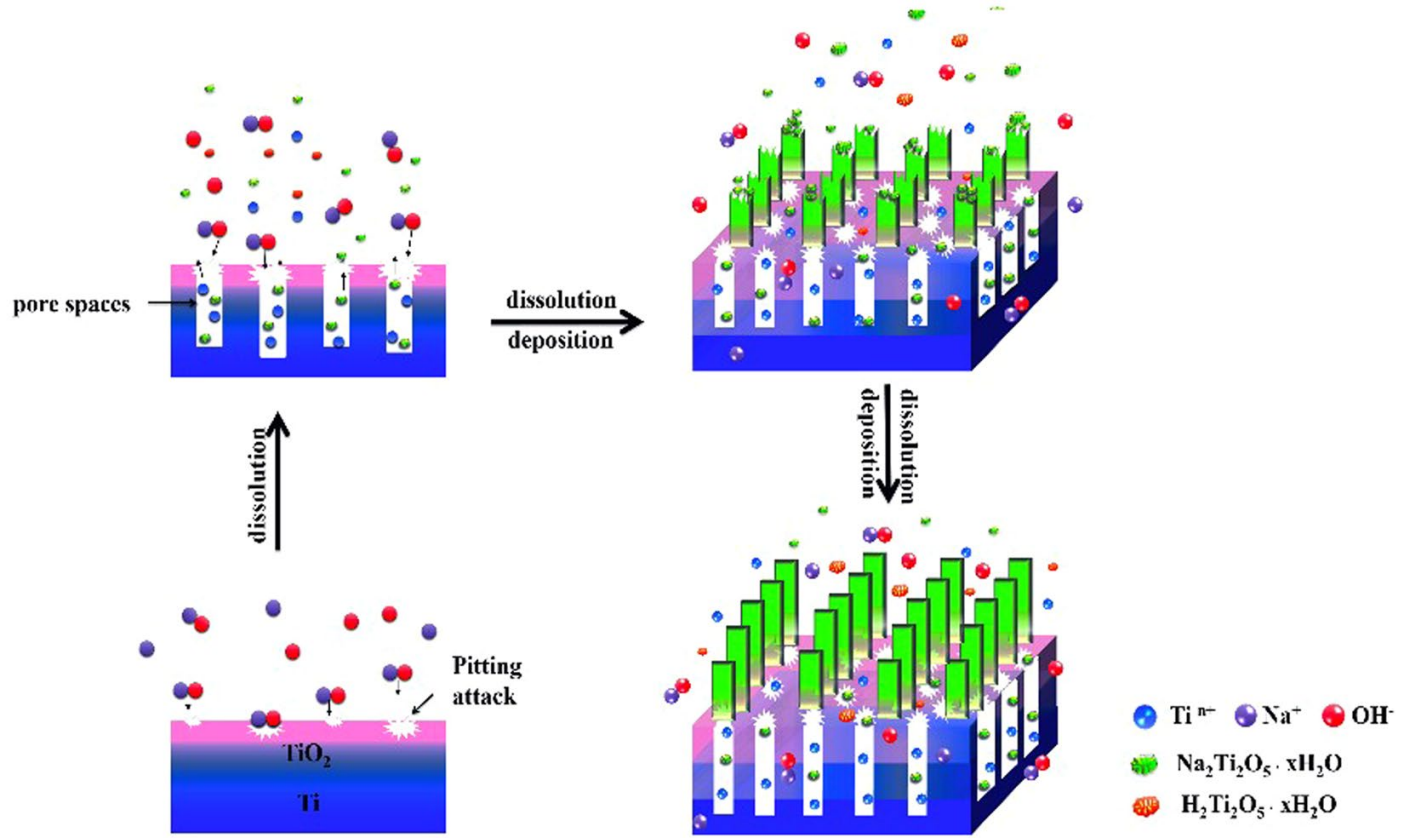
Schematic formation mechanism of flake-like nanostructured titanate layer on Ti substrate.

After stripping the treated surface layer, we can see that both the top region and bottom region of the stripped layer are composed of nanoflakes, and the layer shows a porous structure (Fig. 5). It is obvious that the bottom region is not as interconnected as the top region, i.e., much denser than the top region (Fig. 5a). The thickness of top region and bottom region is about 1.5 μm and 2.5 μm respectively, and the sublayer between them is a corrosion region with a thickness of about 0.5 μm (Fig. 5b). On this basis, we proposed a schematic formation mechanism for the flake-like nanostructured titanate layer on Ti substrate, as shown in Fig. 6. Firstly, NaOH in solution reacted with the titanium oxide layer, resulting in the appearance of random defects (so-called “pitting attack”) and enlarged Ti surface area, which in turn increased the surface free energy and chemical potential^43, 44^. With the progress of reaction, more Na^+^ and OH^−^ ions were incorporated into the active surface and made the reaction more intensively. The continuous occurrence of pitting attack led to large number of pore spaces at the bottom of corrosion region^45^. At the same time, a plenty of Ti, Na and titanate ions spilled from the bottom pores and dissolved in the alkaline solution. When the Ti concentration in solution was close to the dynamic equilibrium in solid/liquid phases, the deposition of sodium titanate occurred, followed by continuous growth upward and downward and final formation of the nanoflakes on Ti surface^46^. According to this hypothesis, the Ti substrate surface might involve in two reactions: Ti dissolution and sodium titanate accumulation. The Ti dissolution caused a relatively dense bottom region and titanate accumulation/deposition resulted in a more porous top region. The flat flakes could curl to curled flakes (like petals) by reaction of the dangling bonds on the surface of titanate flakes with the hydroxide ions in solution, as reported by Chen *el al*.^47^.

### *In vitro* cell behavior

#### Cell morphology

**Figure 7:**
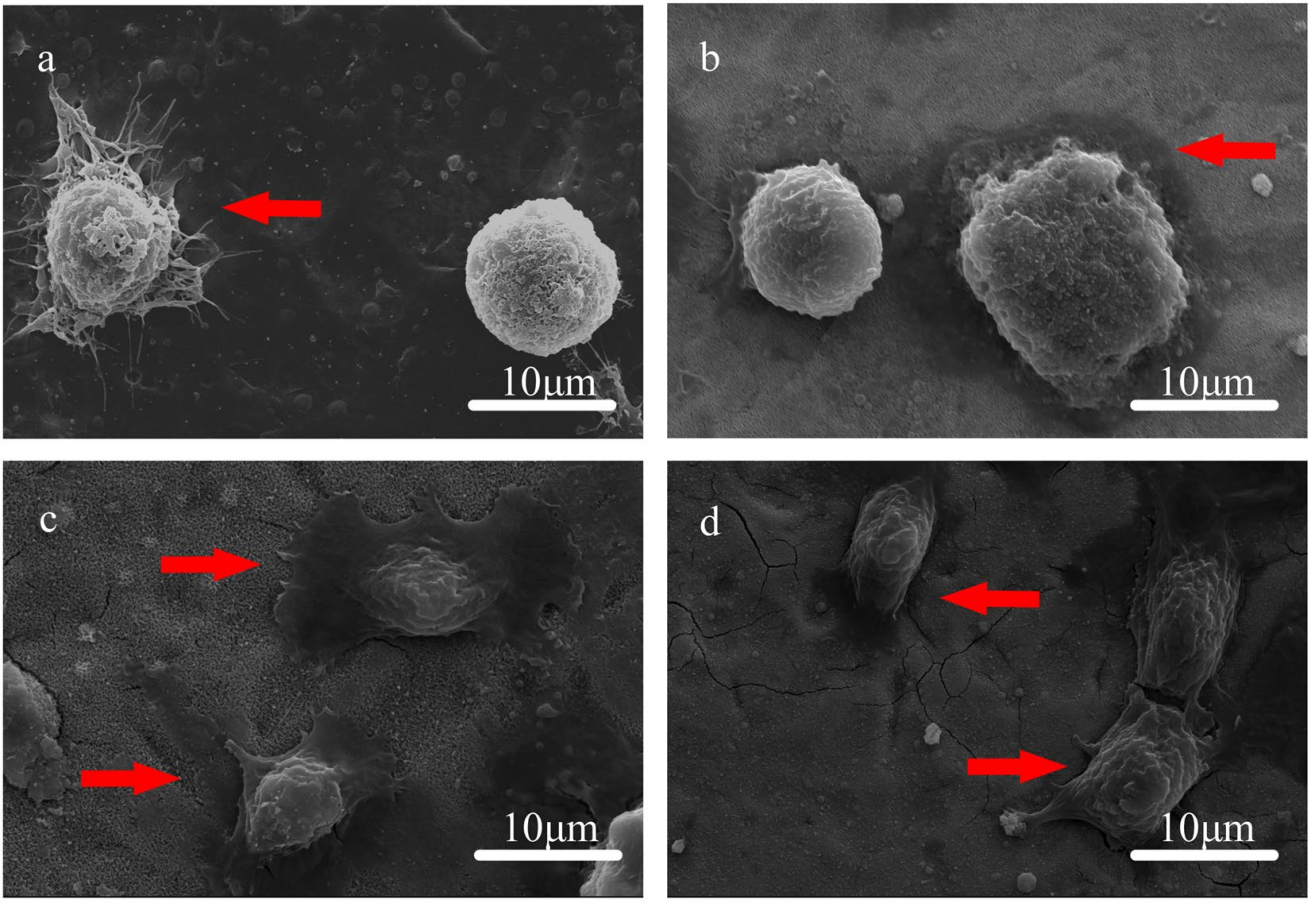
Initial cell adhesion after incubation for 2 h: (**a**) PT; (**b**) T-3; (**c**) T-10 and (d) T-12.5.

The initial adhesion of cells after incubation for 2 h is shown in Fig. 7, where the BMSCs cultured on PT sample show a round shape and almost no spreading presents on the surface, although many filopodial extensions can be observed. In contrast, the spreading of BMSCs cultured on T-3, T-10 and T-12.5 samples are observed, among which the T-10 sample exhibits complete spreading.

#### Cell proliferation

**Figure 8:**
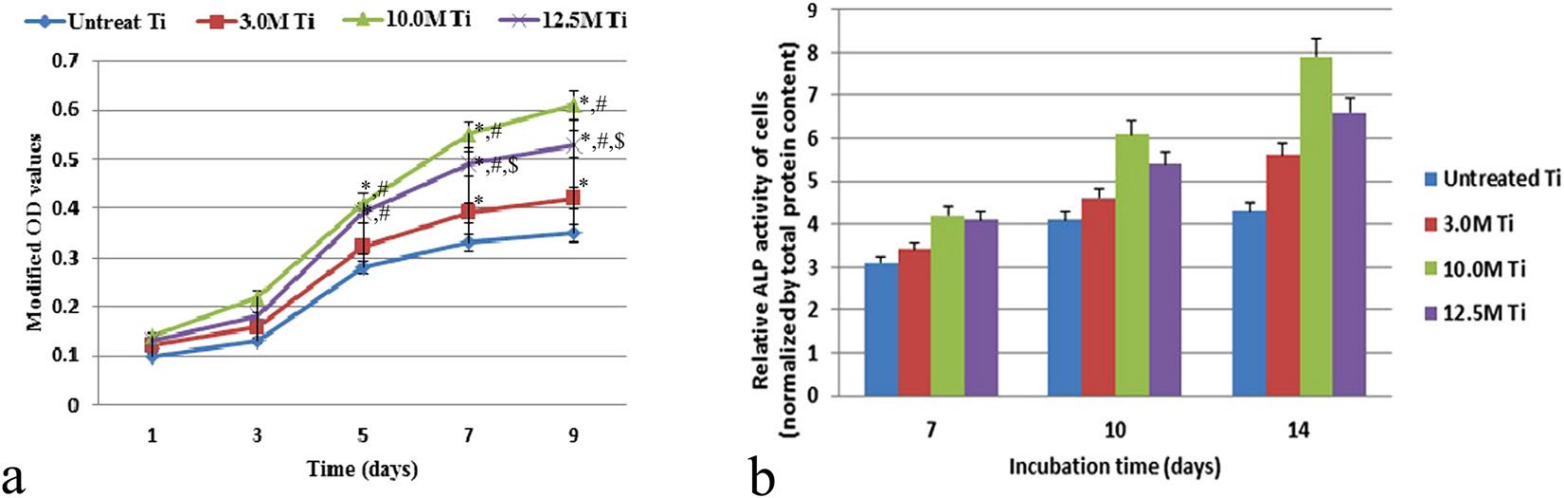
*In vitro* cell behaviours on the PT, T-3, T-10, and T-12.5 samples: (**a**) cell viability; (**b**) ALP activity. ^*^p < 0.05 vs PT, ^$^p < 0.05 vs T-3, ^#^p < 0.05 vs T-10.

The proliferation of BMSCs cultured on the PT, T-3, T-10 and T-12.5 samples is shown in Fig. 8(a). The OD values for all samples increase continuously with the incubation time, indicating a normal growth trend and good cytocompatibility. It should be noted that all treated samples are better than the untreated PT sample, and sample T-10 owns the highest cell proliferation rate than others (P < 0.05). This means surface modification by HPT method, especially the flake-like nanostructured layer on treated Ti surface is beneficial to cell proliferation or growth. The rank order from highest to lowest for cell proliferation is T-10 > T-12.5 > T-3 > PT (P < 0.05).

#### Cell differentition

The relative ALP activity of BMSCs cultured on PT, T-3, T-10 and T-12.5 samples is demonstrated in Fig. 8 (b). It can be seen that the relative ALP activity increases progressively from Day 7 to Day 14, of which the T-10 and T-12.5 samples are significantly higher than the PT and T-3 samples (p < 0.05), and T-10 sample owns the highest value, indicating an enhanced osteogenic differentiation of BMSCs on the treated T-10 surface.

#### Cell seeing and staining

**Figure 9:**
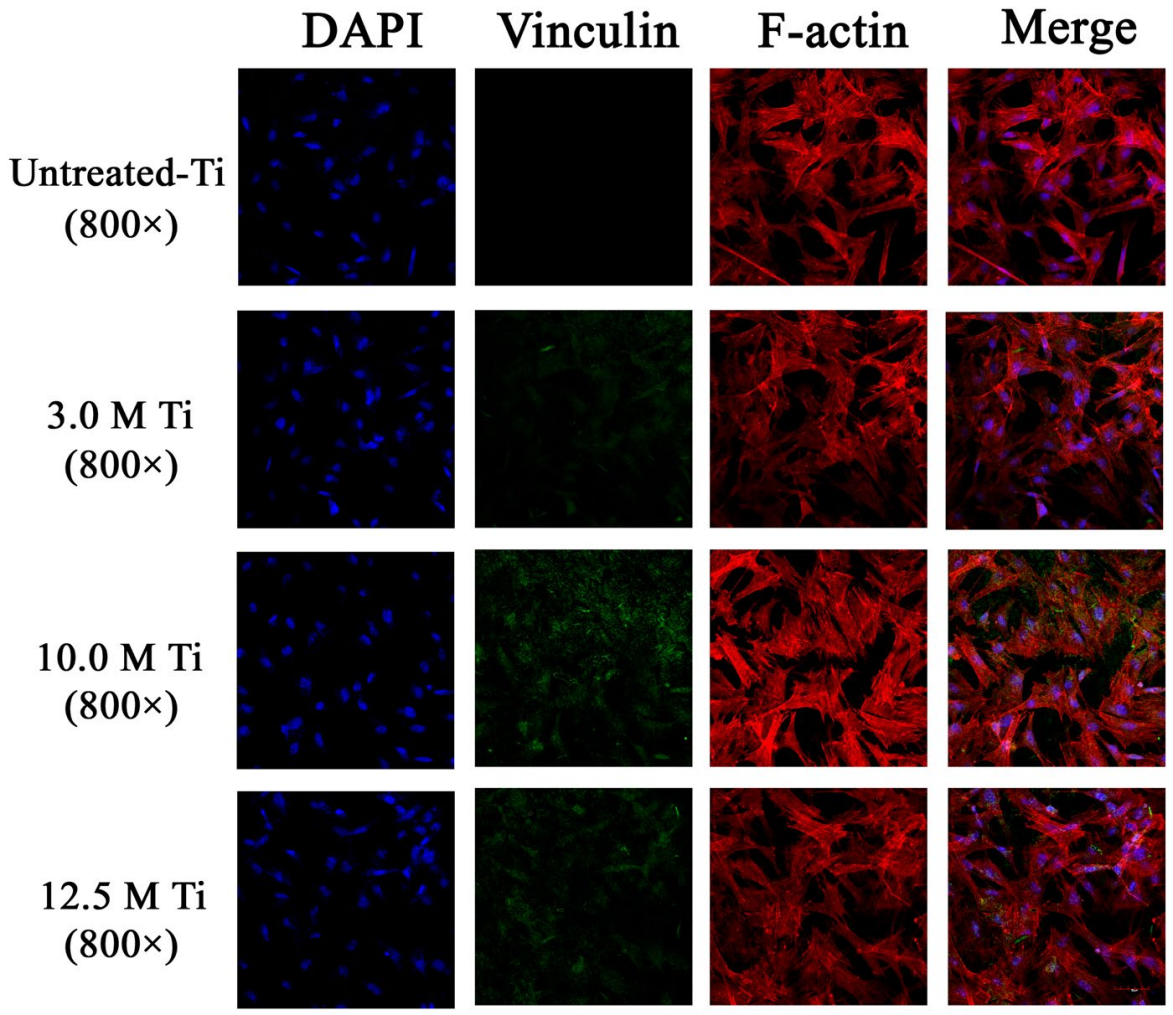
The expression of F-actin and vinculin detected by immunofluorescence assay, and the merged images of F-actin, vinculin and DAPI nuclei on different treated surface.

The cytoskeleton and focal protein adhesion of BMSCs cultured on PT, T-3, T-10 and T-12.5 are shown in Fig. 9. The stained F-actin images indicate that BMSCs on different samples spread very well, with the presence of many F-actin filaments aggregation. Additionally, after 24 h of culture, green fluorescence stained vinculin in BMSCs cultured on T-10 sample is more abundant than other samples. Although T-12.5 sample also displays strong vinculin expression, the difference between the two groups is significant.

#### qRt-PCR of osteogenic factors

**Figure 10:**
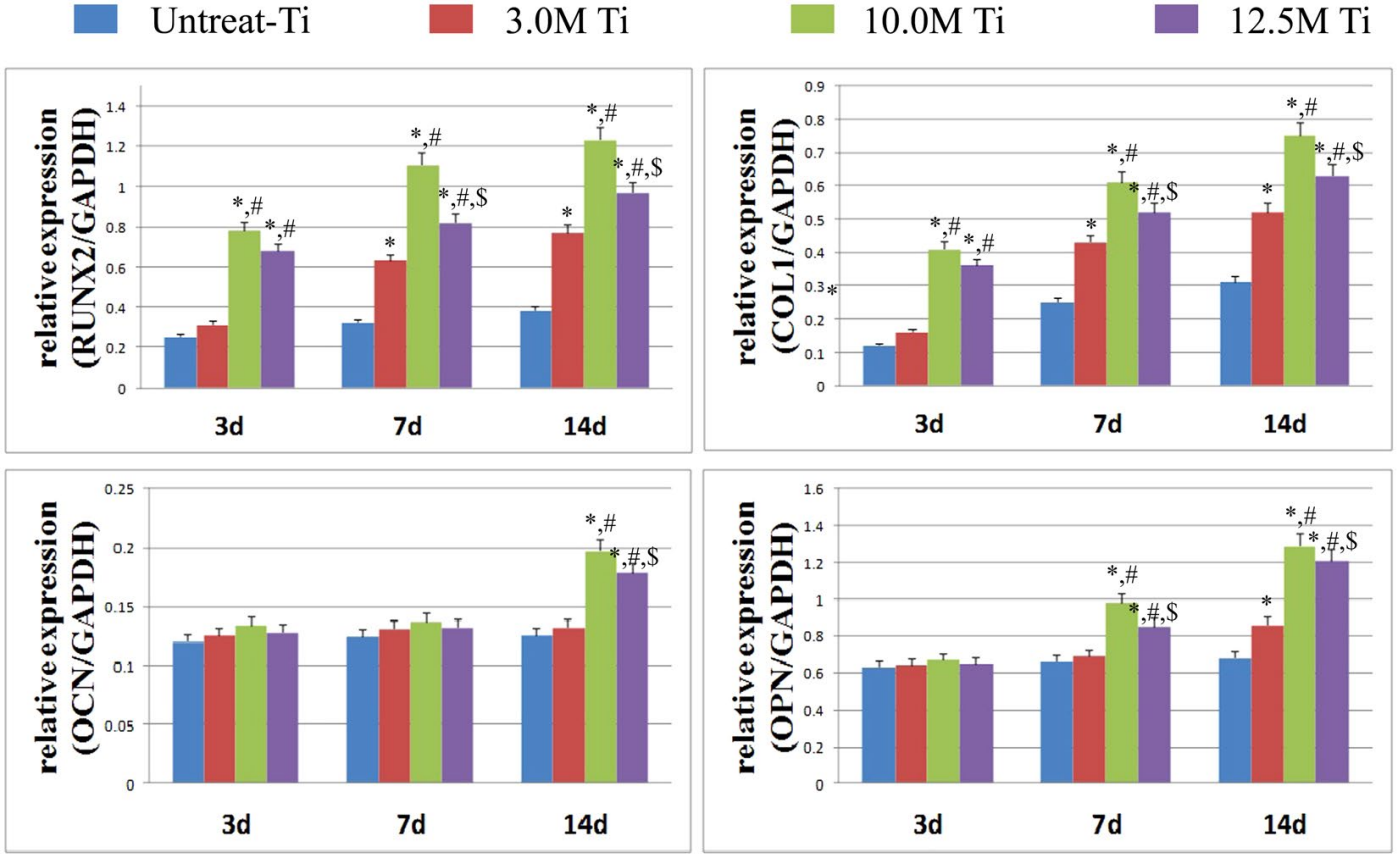
The mRNA expression levels of various osteogenic factors determined by qRT-PCR. *p < 0.05 vs untreated-Ti, ^#^p < 0.05 vs 3.0M-Ti, ^$^p < 0.05 vs 10.0M-Ti.

The mRNA expression levels of various osteogenic factors (Runx2, OCN, OPN, and COL I) were determined by qRT-PCR for PT, T-3, T-10 and T-12.5 samples, as shown in Fig. 10. Obviously, the mRNA expression levels of Runx2 and COL I increase markedly with the culture time, in an order of T-10 > T-12.5 > T3 > PT (p < 0.05). For OCN expression, T-10 and T-12.5 samples show much higher level than T-3 and PT on Day 14. The OPN expressions of T-10 and T-12.5 are also higher than T-3 and PT on Day 7 and Day 14. These results indicate that the T-10 and T-12.5 samples, especially the T-10 sample, can obviously facilitate the early osteogenic transformation of BMSCs.

It can be seen from the results mentioned above, the HPT treatment is a useful method to achieve high surface bioactivity of Ti substrate and enhance the *in vitro* cell-material interactions. The reasons maybe as follows: nano-sized surface treatment can stimulate cell adhesion and rapid osteoblast mineralization as well as accelerate osseointegration *in vivo*. At the same time, the presence of interconnected micro-pores can reduce the relative movement at the bone–implant interface, provide a route for blood circulation and nutrition supply, and achieve a mechanical interlocking, which is particularly beneficial for the biomechanical stability^48, 49^. Furthermore, it has been proved that normal bone trabecula is hierarchical with the presence of micro/nano-structure, which implies that the endosseous implant should have surface features not only on the nanometer scale but also on the micrometer scale^50, 51^. Among all the treated samples, the T-10 sample with a flake-like nanostructured layer possesses relatively the best surface bioactivity, especially in terms of cell adhesion, proliferation and osteogenic differentiation, suggesting its beneficial and promising effect to improve the osseointegration of Ti implants.

## Conclusion

A novel technique called HPT method here was developed to produce nanostructured surface on Ti substrate, which greatly reduced the time-consumption and the manufacturing cost. By carefully controlling the conditions of HPT treatment, we could get different morphological nanostructures on Ti surface. The optimum operation conditions including NaOH concentration and treating time were obtained by orthogonal experiments. which are key factors in determining the formation of surface titanate nanostructure. On this basis, the formation mechanism of flake-like nanostructured titanate layer was simulated and analyzed, revealing that the nanoflake-like layer was formed via an upward and downward co-growth route. HPT treatment could provide an additional driving force for the faster nucleation and growth of nanostructured titanate in alkali solution. In addition, the treated surface could greatly enhance the *in vitro* cell-material interactions and exhibited commendable biocompatibility. Especially, the treated Ti-10 sample displayed the best surface bioactivity, representing an effective way for surface modification of endosseous implants.

## Electronic supplementary material

Supplementary Information
