# Supplementary material for: Surface bioactivation through the nanostructured layer on titanium modified by facile HPT treatment
Source: Sci Rep. 2017 Jun 23;7:4155. doi: 10.1038/s41598-017-04395-0 (PMC5482805; doi:10.1038/s41598-017-04395-0)
Supplement: Supplementary file 1 — Supplementary Information [file 41598_2017_4395_MOESM1_ESM.pdf]

Surface bioactivation through the nanostructured layer on titanium modified by facile HPT treatment

Zhijun Guo[a#], Nan Jiang[b#], Chen Chen[a], Songsong Zhu[b], Li Zhang[a*] and Yubao Li[a*]

a. The Research Center for Nano Biomaterials, Analytical & Testing Center, Sichuan University, Chengdu 610064, China

b. State Key Laboratory of Oral Diseases, National Clinical Research Center for Oral Disease, West China Hospital of Stomatology, Sichuan University, Chengdu, Sichuan, 610064, China

Materials and methods

SBF immersion test

The effects of surface nanostructure by HPT treatment on the bioactivity of Ti substrate were evaluated by immersing the treated Ti samples at 37°C in 24 mL of SBF with ion concentrations neatly equal to those of human blood plasma. The SBF recipe was prepared according to Kokubo's[1] formulation and buffered at pH 7.4 with Tris-hydroxymethyl amino methane and 1.0M HCl at 37°C. Samples were placed in an incubator at 37°C for 1, 4, 7 and 14 days, respectively. To keep the ion concentration constant, SBF solution was refreshed every 2 days. At each time point, the corresponding samples were taken out and gently rinsed with deionized water, then dried in a desiccator for further analysis.

Results

When the NaOH concentration is more than 15M, there are no special structure features present, and just a little round granules are dotted on the Ti surface

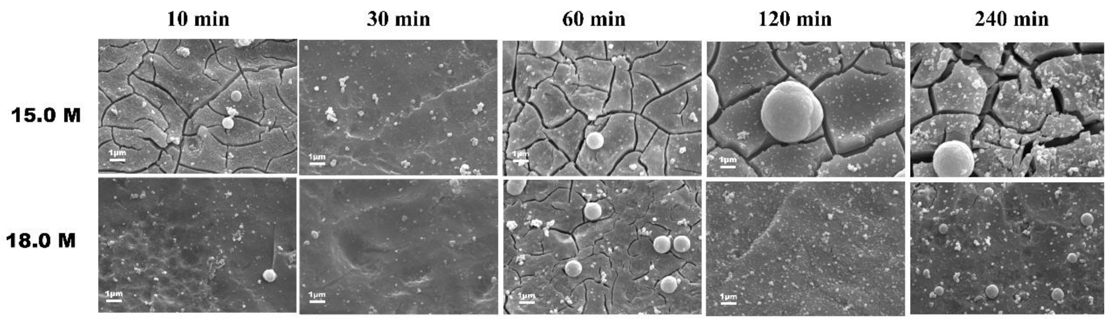

Figure S1. SEM images of Ti surfaces after HPT treatment at 120°C for different time in NaOH solution of 15.0M and 18.0M.

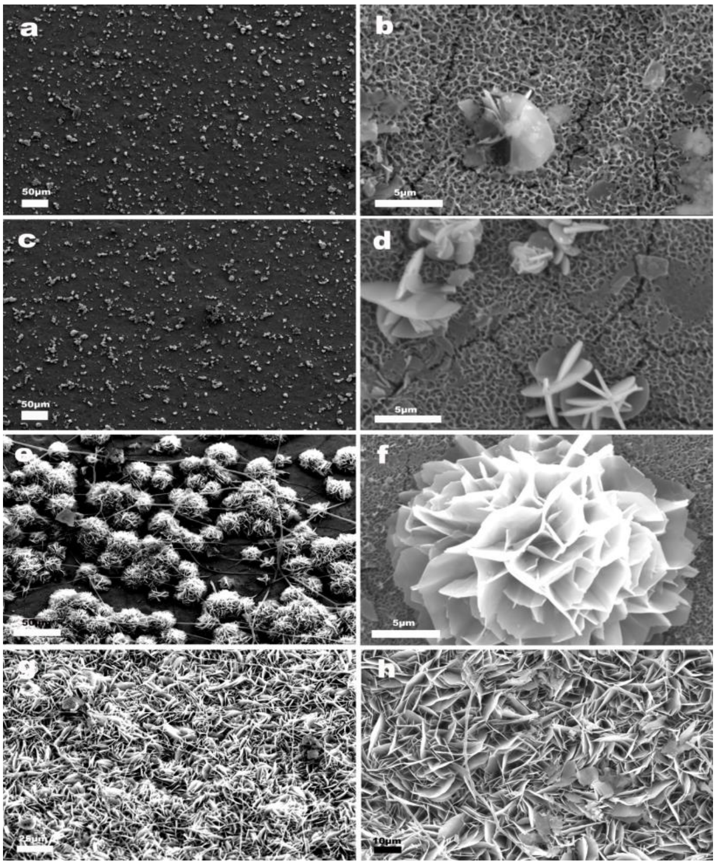

Figure S2. SEM images of Ca-P aggregation and precipitation in SBF for different time on the nanostructured surface of T-10 sample modified by HPT treatment: (a) 1 day, (c) 3 days, (e) 7 days and (g) 14 days; (b), (d), (f) and (h) are images of (a), (c), (e) and (g) with higher magnification, respectively.

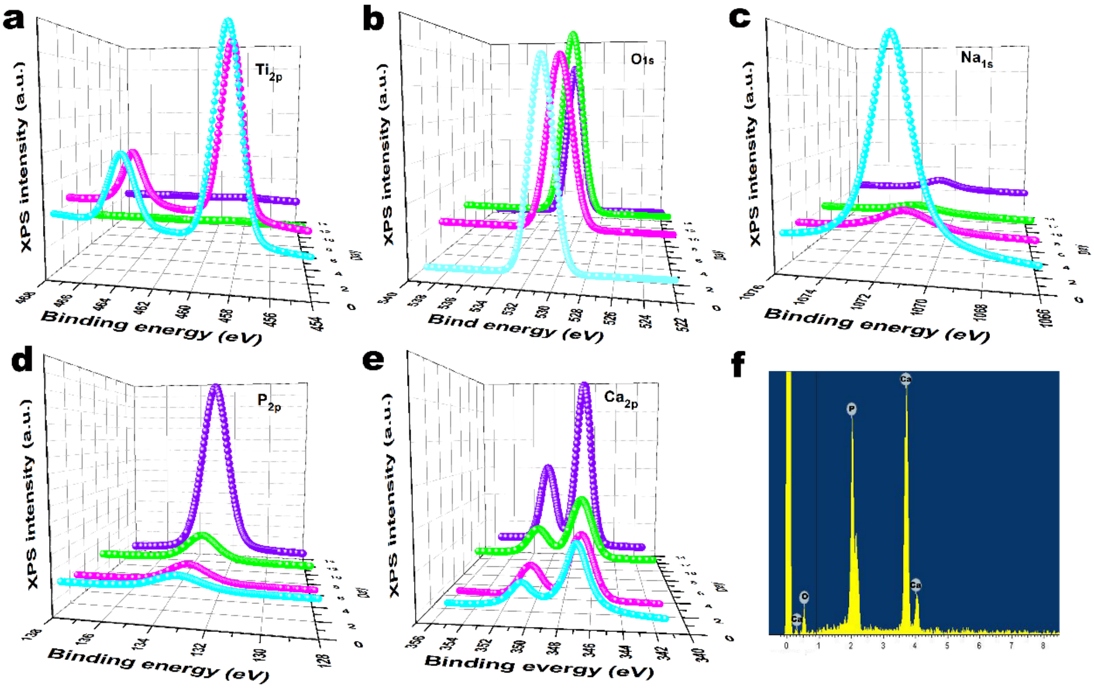

Figure S3. The detected XPS spectra of Ti_{2p} (a), O_{1s} (b), Na_{1s} (c), P_{2p} (d), Ca_{2p} (e), and the EDS element mapping (f) on

the nanostructured surface of T-10 sample after immersion in SBF for 1,3,7 and14 days.

To evaluate the surface bioactivity of nanoflake-like titanate layer on T-10 sample prepared by HPT treatment in 10.0M NaOH solution for 4h, the SBF soaking experiments were carried out to investigate the formation of Ca-P precipitation on the treated Ti-10 sample. As shown in Fig. S2(a, c) after immersion in SBF for 1 and 3 day, plenty of nuclei have deposited on the surface of T-10 sample homogeneously. There are two Ca_{2p} peaks at binding energies of 346.7 eV ($Ca_{2p1/2}$) and 350.2 eV ($Ca_{2p3/2}$) (Fig. S3e) and one P_{2p} peak at 133.1eV (Fig.S3d) appeared in XPS spectra. The Ti_{2p} peaks at 458.5eV ($Ti_{2p1/2}$) and 464.1 eV ($Ti_{2p3/2}$) (Fig. S3a) are attributed to Ti−O bonds[2], while Na_{1s} peak at 1071.3 eV (Fig. S3c) is from the nanoflake-like titanate layer on Ti surface. The detected Ca and P peaks in XPS spectra demonstrate successful deposition of Ca-P aggregations in SBF which is generally regarded as the representative of surface bioactivity. In addition, it has been reported that the nucleation of calcium titanate readily occurred on sphere-like titanate layer.[2-3] The possible reasons is that the formation of Ti-OH is very easy via the exchange of Na^+ ions in sodium titanate with H_3O^+ ions in body fluid.[5, 6-8] The Ti-OH groups are negatively charged [9] and, therefore, likely combine with the positively charged Ca^{2+} ions in body fluid to form an amorphous calcium titanate.[10] Therefore, we can deduce that the deposited nuclei in SBF are mainly composed of Ca-P aggregations and may also contain amorphous calcium titanate.

With the progress of immersion from Day 3 to Day 7, the increase of Ca_{2p} and P_{2p} peak intensity and the decrease of Na_{1s} and Ti_{2p} peak intensity implied that calcium ions accumulated continuously, and the treated Ti surface became more positively charged, which attracted those negatively charged phosphate groups electrostatically to form plenty of amorphous Ca-P nuclei.[10] As shown in Fig. S2(e, f), the nuclei grew up and a large number of nanoflower-like Ca-P spheres with a diameter of 20~30 μm deposited on T-10 surface on Day 7.

After immersion for 14 days, the spherical Ca-P aggregations formed a precipitation layer covering the Ti surface entirely (Fig. S2g). In Fig. S3, the Ca_{2p} and P_{2p} peak intensity further increased markedly, and the Ti_{2p} peaks almost disappeared completely, but small Na_{1s} peak was still present. The EDS element mapping in Fig. S3f also testified that the precipitation layer was mainly Ca-P compound. According to the report of Kim et al,[10] the calcium phosphate precipitate could spontaneously transform into crystalline hydroxyapatite (HA), which is based on the fact that the solubility of HA is much lower than that of other calcium phosphates in body fluid.

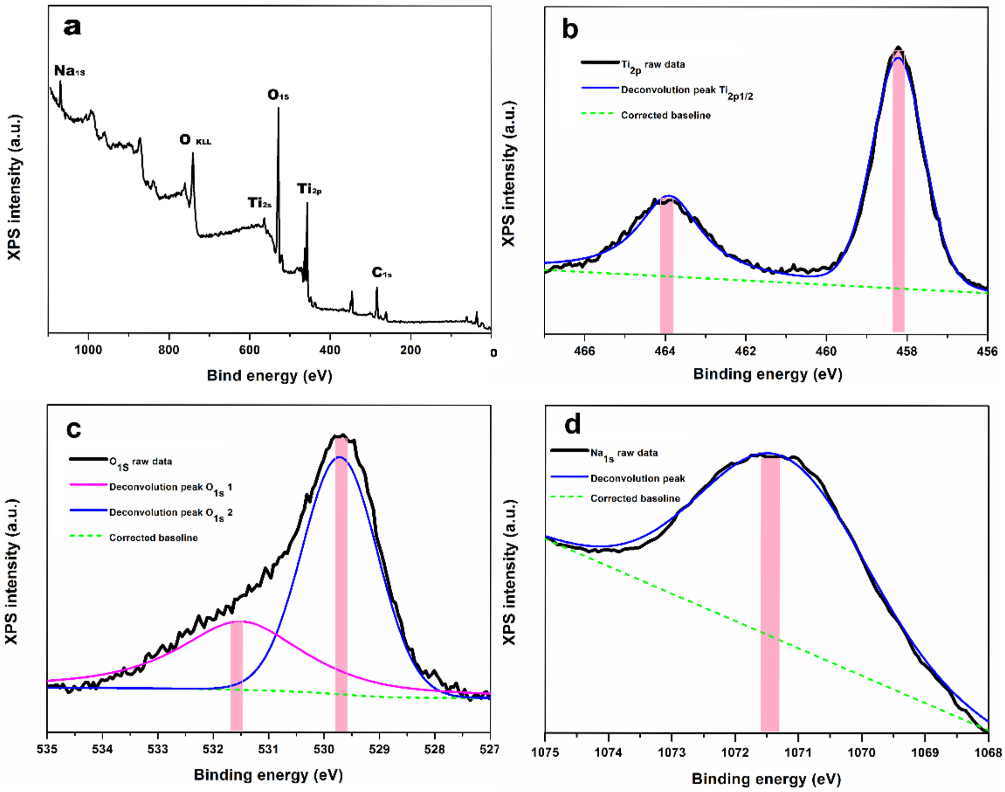

Figure S4. Wide-range survey scan (a) and narrow scans of XPS spectra of Ti2p (b), O1s (c), and Na1s (d) peaks for T-10 sample.

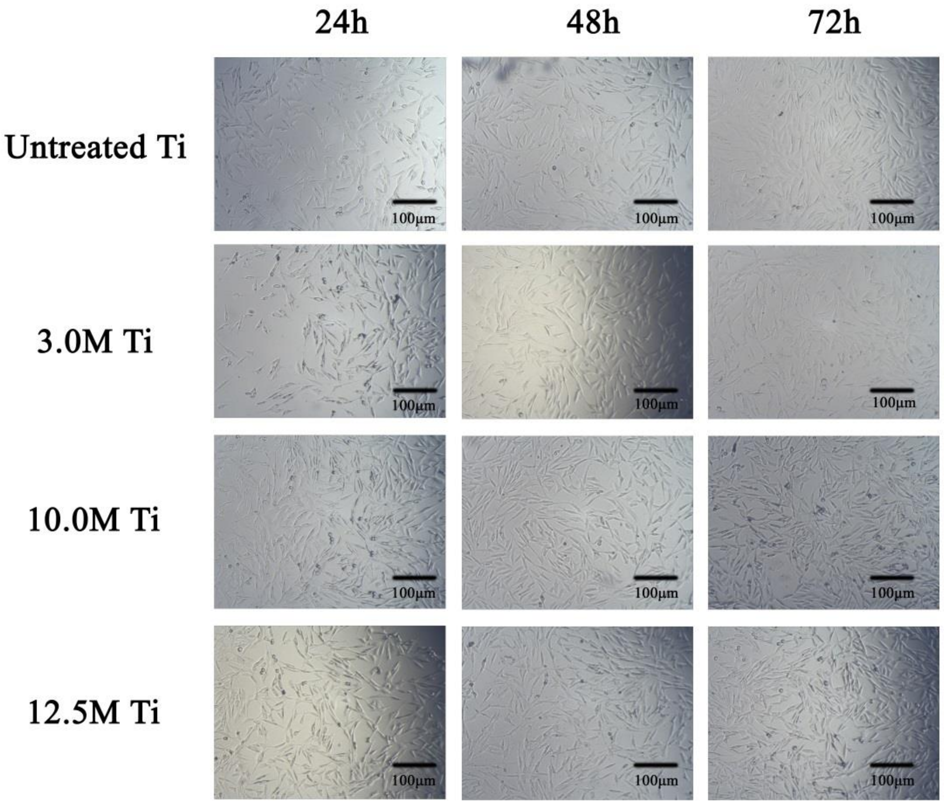

Figure S5. Initial cell proliferation close to Ti substrate observed via optical microscopy

References

1. Kokubo, T., Takadama, H. How useful is SBF in predicting in vivo bone bioactivity? *Biomaterials* **27**, 2907-2915 (2006).
2. Song, H. et al. Preparation and photocatalytic activity of alkali titanate nano materials $A_2Ti_nO_{2n+1}$ (A=Li, Na and K). *Materials Research Bulletin* **42**, 334-344 (2007).
3. Pattanayak, D. et al. Effect of HCl concentrations on apatite-forming ability of NaOH–HCl- and heat-treated titanium metal. *J Mater Sci: Mater Med* **20**, 2401-2411(2009).
4. Sugiyama, N. et al. Bioactive titanate nanomesh layer on the Ti-based bulk metallic glass by hydrothermal–electrochemical technique. *Acta Biomater* **5**, 1367-1373 (2009).
5. Kim, H. M., Miyaji, F., Kokubo, T., Nakamura, T. Preparation of bioactive Ti and its alloys via simple chemical surface treatment. *J. Biomed. Mater. Res.* **32**, 409-417 (1996).
6. Kim, H. M. et al. Graded surface structure of bioactive titanium prepared by chemical treatment. *J. Biomed. Mater. Res.* **45**, 100-107 (1999).
7. Takadama, H., Kim, H. M., Kokubo, T., Nakamura, T. An X-ray photoelectron spectroscopy study of the process of apatite formation on bioactive titanium metal. *J. Biomed. Mater. Res.***55**, 185-193 (2001).
8. Takadama, H., Kim, H. M., Kokubo, T., Nakamura, T. TEM-EDX study of mechanism of bonelike apatite formation on bioactive titanium metal in simulated body fluid. *J. Biomed. Mater. Res.* **57**, 441-448 (2001).
9. Parks, G. A. The Isoelectric Points of Solid Oxides, Solid Hydroxides, and Aqueous Hydroxo Complex Systems. *Chemical Reviews* **65**, 177-198 (1965).
10. Kim, H. M. et al. Surface potential change in bioactive titanium metal during the process of apatite formation in simulated body fluid. *Journal of Biomedical Materials Research Part A* **67**, 1305-1309 (2003).
